# Impairment of electron transport chain and induction of apoptosis by chrysin nanoparticles targeting succinate-ubiquinone oxidoreductase in pancreatic and lung cancer cells

**DOI:** 10.1186/s12263-023-00723-4

**Published:** 2023-03-11

**Authors:** Eman M. Ragab, Doaa M. El Gamal, Tarek M. Mohamed, Abeer A. Khamis

**Affiliations:** Biochemistry Division, Chemistry Department, Faculty of Science, Tanta University, Tanta, 31527 Egypt

**Keywords:** Cancer, Pancreatic, Sirtuin-3, Chrysin, Succinate dehydrogenase

## Abstract

**Background:**

Flavonoids may help ameliorate the incidence of the major causes of tumor-related mortality, such as pancreatic ductal adenocarcinoma (PDAC) and lung cancer, which are predicted to steadily increase between 2020 to 2030. Here we compared the effect of chrysin and chrysin nanoparticles (CCNPs) with 5-fluorouracil (5-FLU) on the activity and expression of mitochondrial complex II (CII) to induce apoptosis in pancreatic (PANC-1) and lung (A549) cancer cells.

**Methods:**

Chrysin nanoparticles (CCNPs) were synthesized and characterized, and the IC_50_ was evaluated in normal, PANC-1, and A549 cell lines using the MTT assay. The effect of chrysin and CCNPs on CΙΙ activity, superoxide dismutase activity, and mitochondria swelling were evaluated. Apoptosis was assessed using flow cytometry, and expression of the C and D subunits of SDH, sirtuin-3 (SIRT-3), and hypoxia-inducible factor (HIF-1α) was evaluated using RT-qPCR.

**Results:**

The IC_50_ of CII subunit C and D binding to chrysin was determined and used to evaluate the effectiveness of treatment on the activity of SDH with ubiquinone oxidoreductase. Enzyme activity was significantly decreased (chrysin < CCNPs < 5-FLU and CCNPs < chrysin < 5-FLU, respectively), which was confirmed by the significant decrease of expression of SDH C and D, SIRT-3, and HIF-1α mRNA (CCNPs < chrysin < 5-FLU). There was also a significant increase in the apoptotic effects (CCNPs > chrysin > 5-FLU) in both PANC-1 and A549 cells and a significant increase in mitochondria swelling (CCNPs < chrysin < 5-FLU and CCNPs > chrysin > 5-FLU, respectively) than that in non-cancerous cells.

**Conclusion:**

Treatment with CCNPs improved the effect of chrysin on succinate-ubiquinone oxidoreductase activity and expression and therefore has the potential as a more efficient formulation than chemotherapy to prevent metastasis and angiogenesis by targeting HIF-1α in PDAC and lung cancer.

**Supplementary Information:**

The online version contains supplementary material available at 10.1186/s12263-023-00723-4.

## Background

Pancreatic ductal adenocarcinoma (PDAC) and lung cancer will surpass colorectal and breast cancer as the second most common cause of cancer-related deaths by 2030 [13]. A comprehensive evaluation of cancer cell metabolism, as described by Warburg, who was granted the Nobel Prize in Medicine in 1931 for his discovery of the oxygen-transferring enzyme of respiration is essential for understanding this illness [26].

The switch from oxidative phosphorylation to aerobic glycolysis arises as a result of metabolic reprogramming and is fundamental for cellular adaption to a hypoxic environment. Hypoxia-inducible factor 1 (HIF-1) is a cellular oxygen sensor that regulates hypoxic responses at a molecular level [30]. An elevated level of HIF-1 has been linked to metastasis, angiogenesis, development of chemo/radioresistance, and overall poor prognosis in cancer patients [48, 55]. Mitochondria are subcellular organelles that play an essential role in the production of ATP, reactive oxygen species (ROS) generation, and the regulation of apoptosis. Succinate dehydrogenase (SDH), frequently referred to as mitochondrial CII, plays two unique roles in mitochondrial metabolism, being a complex ΙΙ of the electron transport chain ETC and an enzyme in the tricarboxylic acid (TCA) cycle. SDHA catalyzes succinate to fumarate oxidation, and electrons produced by this reaction are transferred to the inner-membrane subunits (SDHC and SDHD) through the SDHB iron-sulfur sites and delivered to the ETC quinone pool. The CΙΙ thus has two different enzyme activities: succinate oxidation and ubiquinone oxidoreductase [50].

The activity of SDH is also regulated at the post-translational level through different mechanisms, in particular phosphorylation and acetylation. Thirteen lysines of SDH subunit A are reported to be acetylated, and the loss of SIRT-3, a NAD-dependent deacetylase, results in a decrease of SDH enzymatic activity, indicating that SIRT-3 is an important regulator of SDH activity [6].

Inhibiting tumor-specific abnormalities of mitochondrial metabolism by using a natural product could be an efficient treatment approach for activating the cell death pathway in cancer cells [2]. Chrysin (5, 7-dihydroxyflavone), a natural flavonoid found in a variety of foods and plants, including honey, mushrooms, propolis, and passion flowers, is currently being evaluated for its biologically important anti-inflammatory and anticancer properties, although it has limited effect because of poor solubility and bioavailability [25]. Recent developments in nanotechnology provide a chance to encapsulate anticancer medications in nanocarrier systems. Nanocarriers significantly boost the penetration of biologicals into targeted organs while also allowing for regulated and sustained release [31, 60]. By enhancing their bioavailability, solubility, and stability, and enabling a regulated and extended release, this strategy can assist in overcoming some restrictions on the applicability of flavonoids. This ensures a targeted action, which reduces side effects and increases efficacy [12]. Recent studies showed that the use of a polysaccharide nanocarrier contributes to preserving antioxidant properties and improves the bioavailability of flavonoids in vitro and in vivo [9].Targeting drug to the lungs and pancreas using a nanocarrier seems like a promising idea. It offers great chances to enhance medication therapy both globally and locally [29]. The best method for treating pancreatic and lung cancer is to administer medications directly into those organs [7]. In addition to sustained drug delivery to the pancreas and lungs, prolonged duration of action, lower therapeutic dose, increased patient compliance, and decreased side effects of extremely toxic medications, nano-carriers may also prolong the duration of action [27, 45].

Chitosan (CS) nanoparticles have received considerable attention due to their outstanding physical and biological properties. In our studies, we used chitosan polymer among other materials, due to chitosan being also regarded as a highly versatile polymer. These benefits include its cationic nature, biodegradability, high adsorption capacity, biocompatibility, permeability-enhancing effect, film-forming capabilities, and adhesive features [59].

Chrysin is known to strongly inhibit SDH activity with an increase in ROS generation associated with apoptosis in chronic lymphocytic leukemia (CLL) without affecting the mitochondrial of normal cells by chrysin [47]. Therefore, this study aimed to limit the cellular ability to generate ATP via oxidative phosphorylation by impairing the activity of mitochondrial respiratory chain CΙI, generating superoxide radicals, and inducing apoptosis in cancer cells by inhibiting metastasis and angiogenesis by targeting HIF-1 expression. Our work was to evaluate the effect of chrysin and CCNPs in comparison with that of 5-FLU on the activity and expression of succinate-ubiquinone oxidoreductase and the induction of apoptosis in pancreatic (PANC-1) and lung (A549) cancer.

## Methods

### Chemicals

All analytical grade reagents and chemicals used in this work were purchased from Sigma-Aldrich and were similar to those previously described [44]. Superoxide dismutase (SOD) was purchased from Bio-Diagnostic Co. (Egypt). Annexin V- FITC apoptosis detection kit was purchased from Immunostep Co. (Spain). RT-qPCR reagents, including primers (25 nM), desalt purification, RNA extraction, cDNA synthesis kits, and Evergreen Master mix were obtained from ThermoFisher Scientific (USA).

### Chrysin nanoparticles synthesis

Pure chrysin powder was dissolved in 96% methanol to blend with CS nanoparticles. The chrysin solution was then combined with 0.1% w/v CS solution with stirring [53, 44].

#### Nanoparticle physical characteristics

The entrapment efficiency (EE) of chrysin was measured using a UV/Vis spectrophotometer (Shimadzu, Japan). Relevant calibration curves were established by using supernatants of standard chrysin solutions (10–100 g/mL) [35, 53, 44]. Fourier transform-infrared (FT-IR) analysis was performed to elucidate the structures of pure chrysin and CCNPs [53, 44]. X-ray powder diffraction (XRD) was used to record XRD patterns of chitosan and CCNPs [4, 44]. The zeta potential (ζ) of CCNPs/chrysin was estimated by measuring electrophoretic mobility (*µ*_e_) [34, 44]. The morphology and size of the nanoparticles of CCNPs were measured using a high-resolution transmission electron microscope (TEM) JEOL JEM-2100 [9, 44]. The morphology, size, and shape of the CNNP surface were investigated using scanning electron microscopy (SEM) after gold coating. (TESCAN VEGA 3 SBH model) [32, 44]. In vitro drug release was studied as previously described [15, 44]. Briefly, the amount of chrysin released from microspheres at a given time was determined through the following equation:

1 $$\bf Chrysin\;release\;(\%)\;=\;Amount\;of\;chrysin\;released\;from\;microspheres\;/\;Total\;loading\;amount\;of\;chrysin\;in\;microspheres\;\times\;100$$

### In vitro cell viability studies using the MTT assay

Human Pancreatic adenocarcinoma (PANC-1), human pulmonary adenocarcinoma (A549), and non-cancerous (Primary Lung normal fibroblasts) cells were obtained from American Type Culture Collection (ATCC, NY, USA) via Vacsera (Egypt). The experiments will be monitored by the Egyptian Ethical Committee of Tanta University’s Faculty of Science (IACUC-SCI-TU-0165). They were cultured according to the standard protocols, and MTT assays were used to assess cell proliferation (viability) [8, 44]. Cells were counted and then re-seeded in 96-well plates to a final concentration of 1 × 10^5^ cells/mL (3 × 10^4^ cells/well). Cells were then treated with different concentrations of chrysin (12.5–100 μM), CCNPs (12.5–100 μΜ), chitosan nanoparticles (CNPs) (500–2000 μg/mL), and 5-FLU as parallel control (3.13–100 μM as triplicate for each concentration and the cells were cultured for 48 h. After drug treatment for 48 h, the culture medium was gently washed twice with ice-cold PBS,At the end of incubation, 10 μl of 12 mM MTT stock solution was added to each well. The plate was then incubated for 4 h at 37 °C. The 96-well microplates were analyzed as previously described [42, 44].

### Isolation of crude mitochondria from cancer cell lines

Mitochondria were isolated from 60 × 10^6^ A549, PANC-1, or non-cancerous cells as previously described [62, 44].

#### Determination of SDH activity by MTT assay

The MTT test was used to evaluate the SDH activity as previously described [44]. Briefly, a mitochondrial suspension (50 μL) was incubated with a range of concentrations based on the IC_**50**_ of chrysin (57, 93, and 129.11 μg/mL), CCNPs (14, 20, and 155.6 μg/mL), or 5-FLU (5.8, 2.8, and 8.07 μg/mL) in A549, PANC-1, and non-cancerous cell lines. Then, 20 mM succinate, 5 mM MgCl_2_, 2 mM KCN, 0.4 mg/mL rotenone, and 0.02 mg/mL antimycin were added, with 50 mM phosphate buffer pH 7.4, and 0.25 mg/mL MTT was added. Each assay was incubated for 30 min at 37 °C. Next, 50 μL of DMSO was used to dissolve formazan crystals. The absorbance was measured with an ELISA reader at 570 nm [51].

#### Determination of succinate-ubiquinone oxidoreductase activity (CII)

The mitochondrial suspension (50 μL) obtained from PANC-1, A549, and non-cancerous cells were incubated with IC_50_ concentration ranges of chrysin (93, 57, and 129.11 μg/mL), CCNPs (20, 14, and 155.6 μg/mL), or 5-FLU (2.8, 5.8, and 8.07 μg/mL). Cells were then incubated in a medium containing 12.5 mM potassium phosphate buffer (pH 7.4), 5 mM MgCl_2_, and 20 mM of succinate at 30 °C for 10 min. Then, 2 mM KCN (to inhibit complex), 0.02 mg/mL antimycin A (to inhibit complex III), 0.4 mg/mL rotenone, and 0.05 mM 2,6 DCPI was added, and a baseline rate was recorded for 3 min. The reaction started with the addition of 0.065 mM ubiquinone. The enzyme-catalyzed reduction of DCPI was measured for 5 min by following the decrease in absorbance at 600 nm [56, 39, 44].

#### Determination of protein content

The mitochondrial protein content was detected using the Bradford reagent [22].

### Activity of mitochondrial manganese-superoxide dismutase

The addition of superoxide dismutase inhibited the reduction of nitro blue tetrazolium (NBT) with NADH mediated by phenazine methosulfate (PMS) under aerobic conditions. This observation demonstrated that the superoxide anion radical (O2^**−**^), which is produced during the reoxidation of reduced PMS, was involved in the reduction of NBT. Superoxide dismutase also inhibited the reduction of NBT coupled to the D-amino acid oxidase-PMS system under aerobic conditions. Trypsinized cells were mixed with a buffer containing 1.2 mL sodium pyrophosphate, 0.1 mL PMS, and 0.3 mL NBT, to a total volume of 2.8 mL with water. The reaction was triggered by adding 0.2 mL NADH after 90 s of mixing at 30 °C as previously described [43].

### Mitochondrial swelling

Mitochondrial swelling was determined based on the absorption decrease at 540 nm for 10 min as previously described [65, 44].

### Flow cytometry analysis of apoptosis.

Annexin V-FITC apoptosis kit (Immunostep, Spain) was used to measure apoptosis. Cells (300 × 10^3^) were grown in a T-25 culture flask for 48 h and then treated with the IC_50_ of chrysin, CCNPs, or 5-FLU for 48 h. The culture medium was then gently washed out of flasks twice with ice-cold PBS and resuspended with an annexin-binding buffer at 1 × 10^6^ cells/mL. For each cell suspension (100 μL), 5 μL of Annexin V-FITC and 5 μL propidium iodide (PI) were added. Cells were incubated at room temperature (25 °C) for 15 min in the dark. Annexin binding buffer was then added to 1 × (400 μL), and the samples were analyzed. This test is simple and discriminates between intact (FITC/PI^−^), apoptotic (FITC + /PI^−^), and necrotic cells (FITC + /PI^+^) [21].

### Measurement of gene expression with RT-qPCR in A549, PANC-1, and normal cell lines treated with chrysin, CCNPs, or 5-FLU

Using commercial kits (Thermo Scientific Gene JET RNA Co, #k0731, America) total RNA was extracted from treated cells. PANC-1, A549, and non-cancerous cells were treated with IC_50_ concentration of chrysin, CCNPs, and 5 FLU for 48 h. The lysate was mixed with ethanol in a purification column. The reverted cDNA reverse transcriptase was determined with the available commercial kits (cDNA Synthesis Kit Thermo Co, Bio-6505, America). The level of cDNA was measured by Nanodrop. Quantitative PCR of SDH subunits C and D, SIRT-3, and HIF was performed using a Rotor gene 5 plex (QIAGEN) with QIAGEN two-step SybrGreen. The GAPDH gene was used as an internal reference. Primers were designed using BLAST (https://blast.ncbi.nlm.nih.gov/Blast.cgi) (Table 1) [46].

**Table 1:** Forward and reverse primers of 5 genes (1) Housekeeping gene GAPDH, (2,3) SDH subunit C, D. (4) Sirtuin-3 (5) HIF

Gene	Forward primer (^/^5 ––– ^/^3)	Reverse primer (^/^5 ––– ^/^3)	Size (bp)
**GAPDH**	GATTCCACCCATGGCAAATTC	CTGGAAGATGGTGATGGGATT	87
**SDHC**	GATGGAGCGGTTCTGGAATAA	CATGGGAAGAGACCAACTGTAG	85
**SDHD**	CATTTCTTCAGGACCGACCTATC	AACTTGTCCAAGGCCCAAT	88
**Sirtuin3**	AGGGACGATGATGTAGCTGA	GGCGATCTGAAGTCTGGAATG	110
**HIF**	GTCTGCAACATGGAAGGTATTG	GCAGGTCATAGGTGGTTTCT	103

### Statistical analysis

Statistical analyses were performed using GraphPad Prism, version 6 software. Results are expressed as mean ± standard deviation. Two-one-way ANOVA was used to identify significant differences between the two groups. The level of significance was set at *p* ≤ 0.05 (*p ≤ 0.05, **p ≤ 0.01, ***p ≤ 0.001, ****p ≤ 0.0001).

## Results

### Nanoparticle physical characteristics.

#### EE (%) and ultraviolet (UV) analysis

The EE of chrysin was found to be 92.63 ± 1.30% with a distinctive peak in UV analysis of 348 nm as previously shown [44].

#### FT-IR and XRD analysis

We compared the functional groups in CCNPs with those in the individual components. Bands that are distinctive for chrysin showed C–C, C-O, and C–O–C stretching vibrations at 520, 720, and 888 cm^−1^, respectively. The C = O group was represented by the peaks at 1533 and 1653 cm^−1^, whereas the OH group was represented by the band at 3455 cm^−1^. Chitosan has a signature band at 1080, 1648, and 2889 cm^−1^, indicating the presence of the CH_2_ group, the amide band, and the C–H stretch, respectively. In XRD, chrysin showed characteristic peaks at 12˚, 14.4˚, 17.23˚, 20.18˚, 22.03˚, 24.64˚, and 27.36˚. In XRD for CCNPs, there was a shift in peaks so these appeared at 11.2˚, 32.2˚, and 19.6˚, 27.6˚, 31.96˚ [44].

#### Zeta (ζ) potential measurements

As previously described the CS nanoparticles have a positive zeta potential as a consequence of the cationic features of the chitosan molecule between + 35.5 to + 77 mV [44].

#### Particle size measurements (TEM) and SEM analysis

As already stated in our recent analysis, the nanoparticles were found to be spherical with an average particle size of 49.7 ± 3.02 nm, and SEM analysis was performed to analyze chrysin nanoparticle size, surface morphology, and homogeneity. The shape and surface morphology are shown in [44].

#### In vitro release study

In vitro drug release was evaluated in a medium containing PBS at 37 °C with a pH of 7.4 for stability. Chrysin's release was then followed with time. Initially, the kinetic drug release of CCNPs was observed in the first two hours followed by a constant release up to 8 h as previously described [44].

### Cytotoxic effect of chrysin, CCNPs, CNPs, and 5-FLU on A549, and PANC-1 and non-cancerous cell lines after 48 h incubation.

**Fig. 1:**
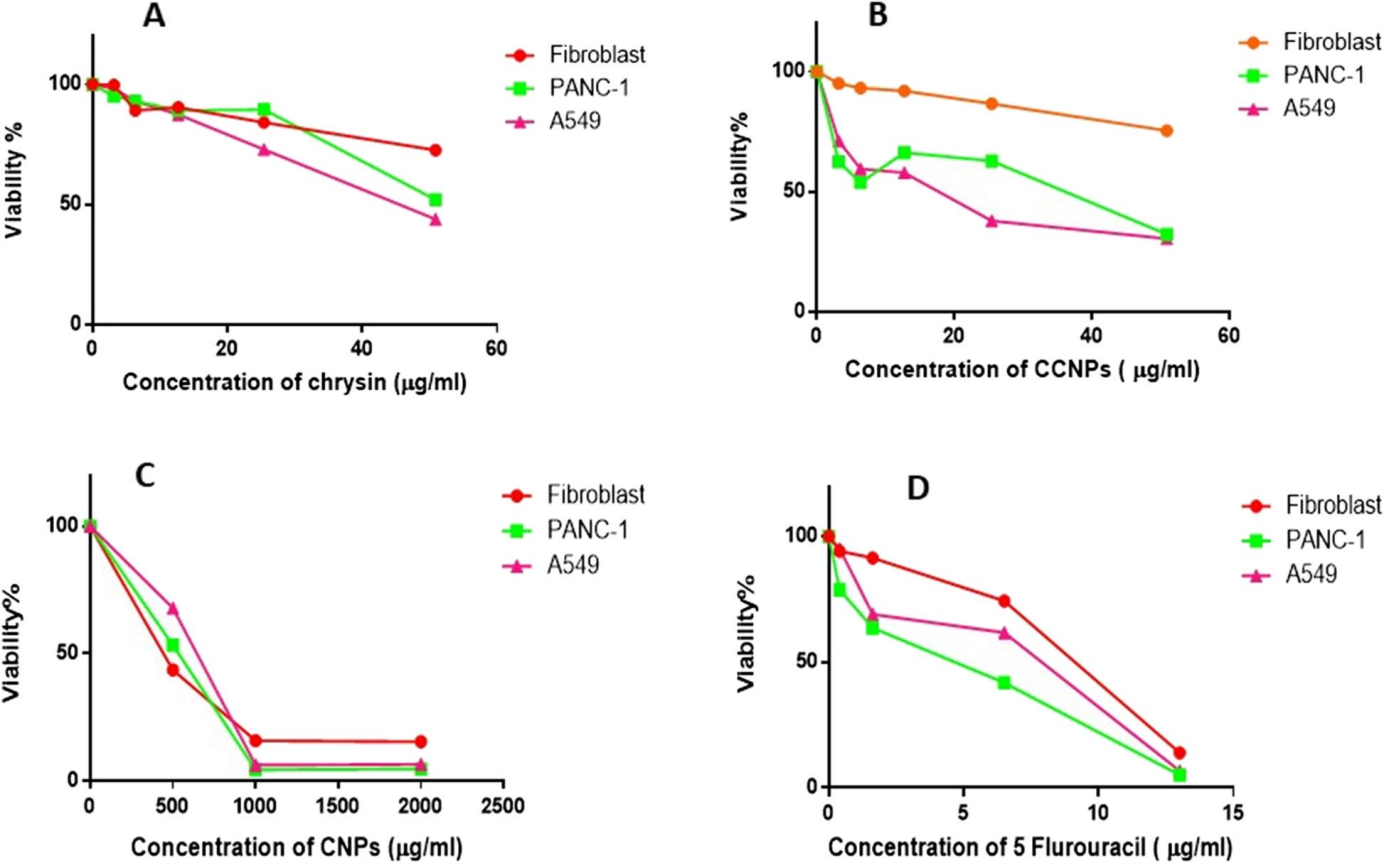
Effect of (**A**) chrysin, (**B**) CCNPs, (**C**) CNPs, and (**D**) 5-FLU on Normal, A549, and PANC-1 cells viability by the MTT assay after 48 h treatment

In the present study, systematic experimental steps in order to determine the potential cytotoxicity of the drug at different concentrations by MTT assay. Cell proliferation was determined using an MTT assay and compared with that obtained using 5-FLU as a positive control. The viability of cancer cells significantly decreased (*p < 0.01) with increasing treatment concentrations (Table 2 and Fig. 1) compared with that in the untreated cells.

**Table 2:** IC_50_ of chrysin, CCNPs, CNPs, and 5-FLU on non-cancerous, A549, and PANC-1 cell lines for 48 h

IC_50_ Concentration of treatment (μg/mL)	Cell lines		
	**Normal**	**A549**	**PANC-1**
**Negative control (untreated cells)**	100 ± 0	100 ± 0	100 ± 0
**Chrysin**			
** mean ± SEM**	129 ± 0.00849	57 ± 0.02	93 ± 0.004
*p value	*p* < 0.01	*p* < 0.01	*p* < 0.01
**CCNPs**			
** mean ± SEM**	156 ± 0.00849	14 ± 0.014	20 ± 0.012
*p value	*p* < 0.01	*p* < 0.01	*p* < 0.01
**5 FLU**			
** mean ± SEM**	8.07 ± *0.025*	5.8 ± 0.05	2.8 ± 0. 13
*p value	*p* < 0.01	*p* < 0.01	*p* < 0.01
**CNPs**			
** mean ± SEM**	311.1 ± 0.013	394 ± 0. 05	285 ± 0. 005
*p value	*p* < 0.01	*p* < 0.001	*p* < 0.0001

^*^p value versus the negative control (untreated cells)

### Influence of chrysin, CCNPs, and 5-FLU on succinate-coenzyme Q oxidoreductase activity in A549, PANC-1, and non-cancerous cell lines

#### Determination of SDH activity by MTT assay

**Fig. 2:**
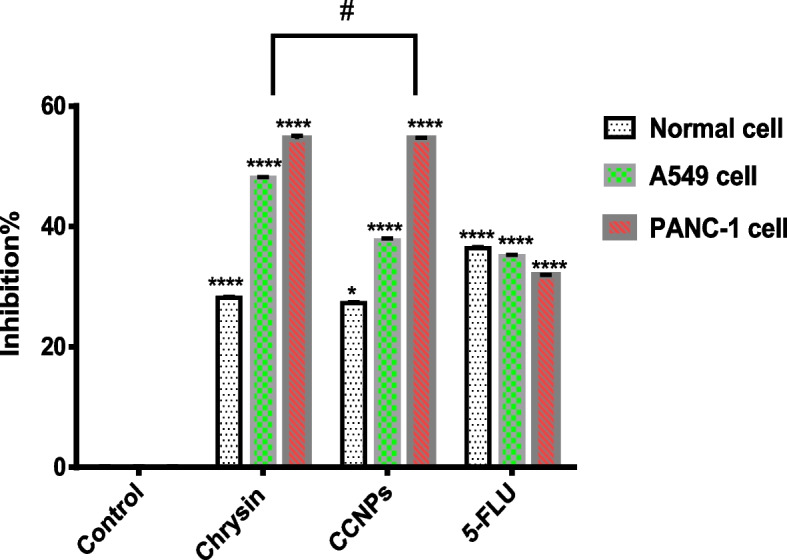
Effect of all drugs on SDH activity in non-cancerous and cancer cell lines. Where ****p < 0.0001, ***P < 0.001, **P < 0.01, *P < 0.05, # for non- significant against each other

Exposure of chrysin, CCNPs, and 5-FLU at concentrations equal to their IC_50_ value caused a significant decrease (*p < 0.0001) in SDH activity as measured by the MTT assay in non-cancerous and cancerous cell lines; CCNPs produced the lowest effect on SDH activity in non-cancerous, whereas 5-FLU produced a significant decrease (5-FLU < chrysin < CCNPs). In A549 and PANC-1 cells, chrysin produced a significant (*p < 0.0001) decrease compared to 5-FLU but has the lowest effect on SDH activity (chrysin < CCNPs < 5-FLU) (Fig. 2) [44].

#### Determination of SDH–Coenzyme Q oxidoreductase activity (CII)

**Fig. 3:**
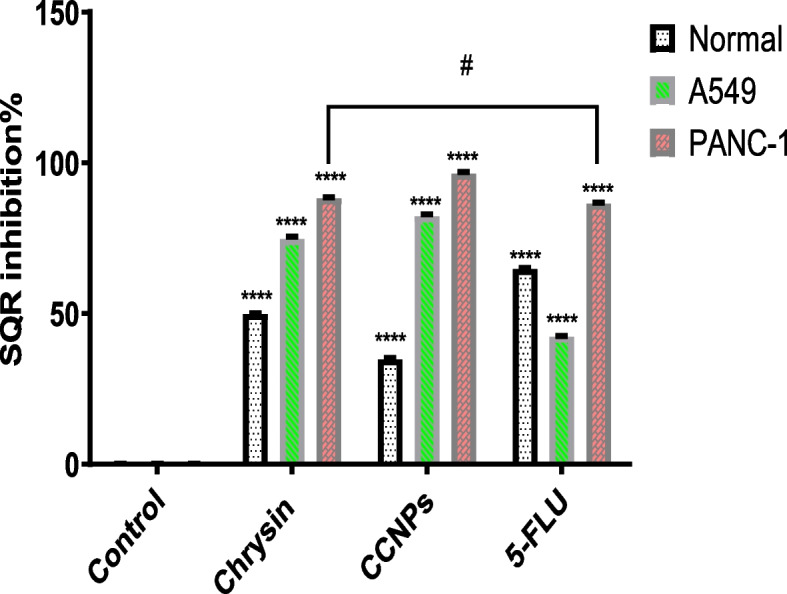
Effect of all drugs on SQR activity in non-cancerous, and cancer cell lines, where ****p < 0.0001, ***P < 0.001, **P < 0.01, *P < 0.05, # for non-significant against each other

The results have demonstrated as previously mentioned exposure to chrysin, CCNPs, and 5-FLU at concentrations equal to their IC_50_ value caused a significant (*p < 0.0001) decrease in succinate–coenzyme Q oxidoreductase activity as measured by the DCPI dye in cancer and non-cancerous cell lines. In non-cancerous cells, 5-FLU showed a significant decrease in enzyme activity but chrysin and CCNPs had the lowest effect on ubiquinone reductase (5-FLU < chrysin = CCNPs) However, in A549 and PANC-1 cells, CCNPs produced a significant (*p < 0.0001) decrease in activity, whereas 5-FLU treatment resulted in the lowest decrease in SDH activity CCNPs < chrysin < 5-FLU) (Fig. 3).

### Effect of 48 h administration of chrysin, CCNPs, and 5-FLU on SOD activity in A549, PANC-1, and non-cancerous cell lines

**Fig. 4:**
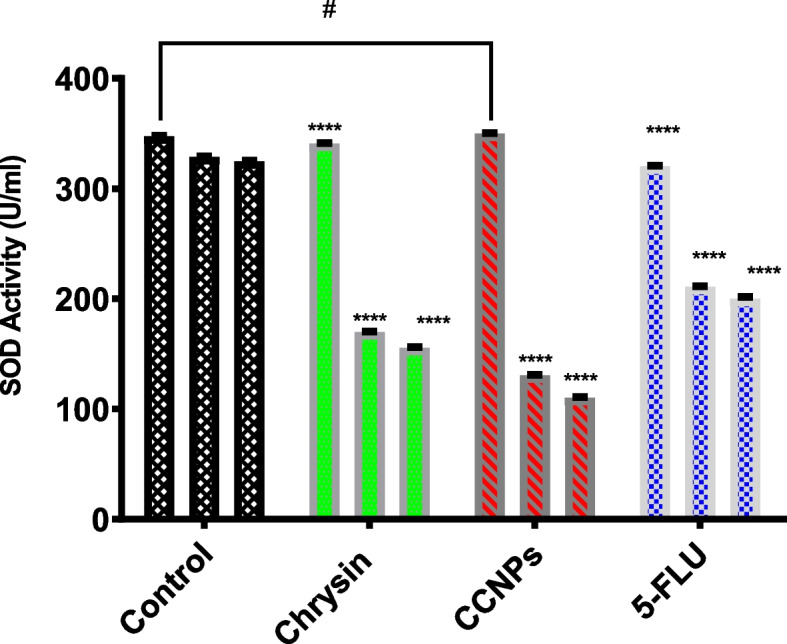
Effect of 48 h administration of chrysin and CCNPs on superoxide dismutase (SOD) activity in non-cancerous cell lines, PANC-1 and A549 cancer cell lines. Where ****p < 0.0001, ***P < 0.001, **P < 0.01, *P < 0.05, # for non-significant against control and each other

We then studied the effect of chrysin, CCNPs, and 5-FLU on the superoxide dismutase activity in cells to indicate the generation of free superoxide radicals. Chrysin and CCNPs indicated a significant (*p < 0.001) decrease in SOD activity in a time-dependent manner following the administration of their IC_50_ dose to A549 and PANC-1 cells (Fig. 4).

### Mitochondria swelling

**Fig. 5:**
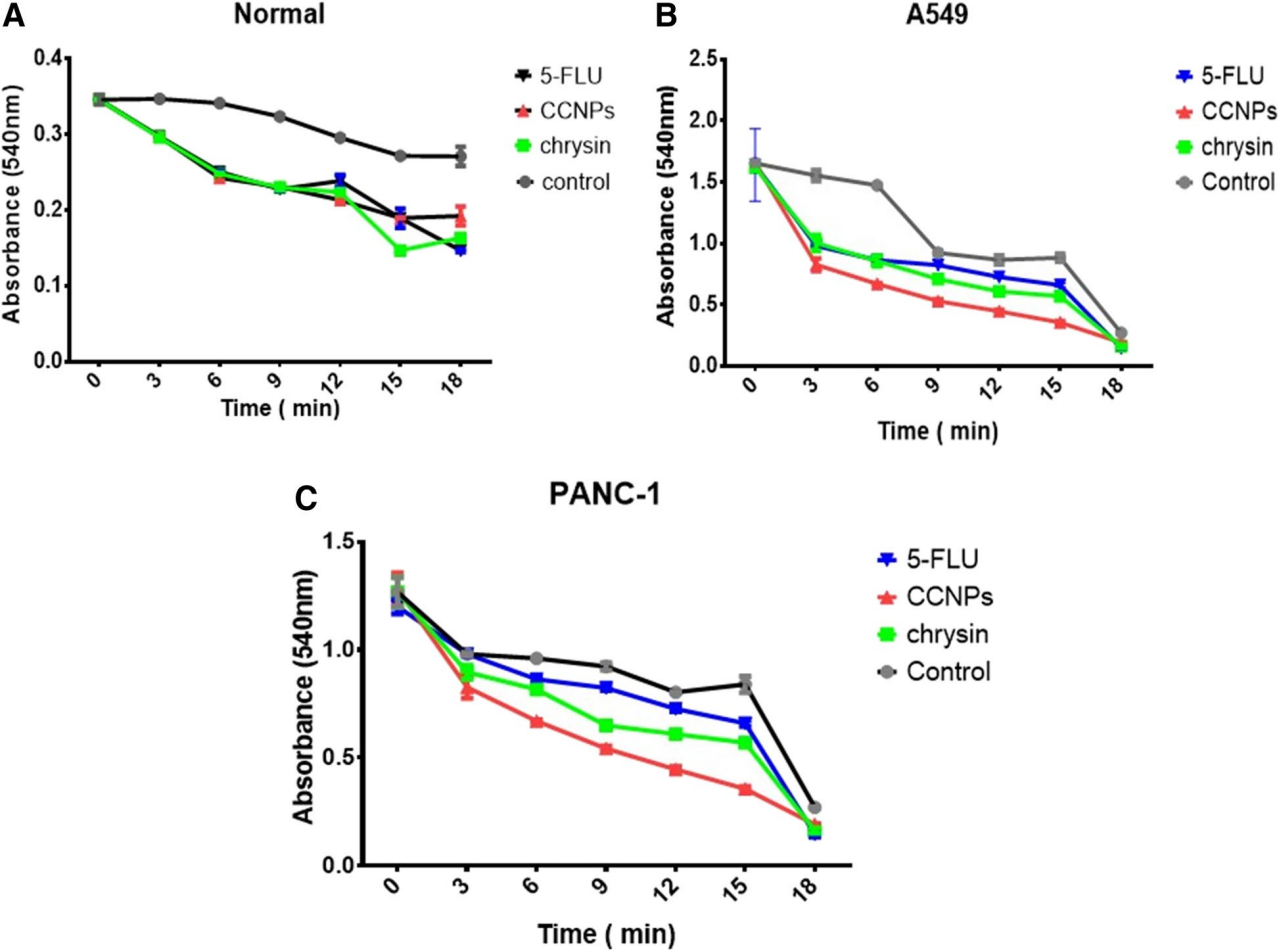
The effect of IC_50_ concentrations of all drugs on the mitochondrial swelling level in the normal (**A**) A549 (**B**) and PANC-1 (**C**). Data are shown as mean SEM and cancer (*n* = 3). A two-way ANOVA test was carried out. ** *P* < 0.01, ***P < 0.001 and ****P < 0.0001, # for non-significant versus the corresponding control group

We found there was a significant (*p ≤ 0.0001) increase in mitochondria swelling in A549 and PANC-1 cells following CCNP treatment. Mitochondrial swelling in the two cancer cell lines induced by CCNPs was greater than that produced by chrysin or 5-FLU (CCNPs > chrysin > 5-FLU) compared with swelling in untreated cells. All treatments produced significant changes in swelling in non-cancerous cells (*p ≤ 0.0001) (Fig. 5).

### Apoptosis analysis by flow cytometry

**Fig. 6:**
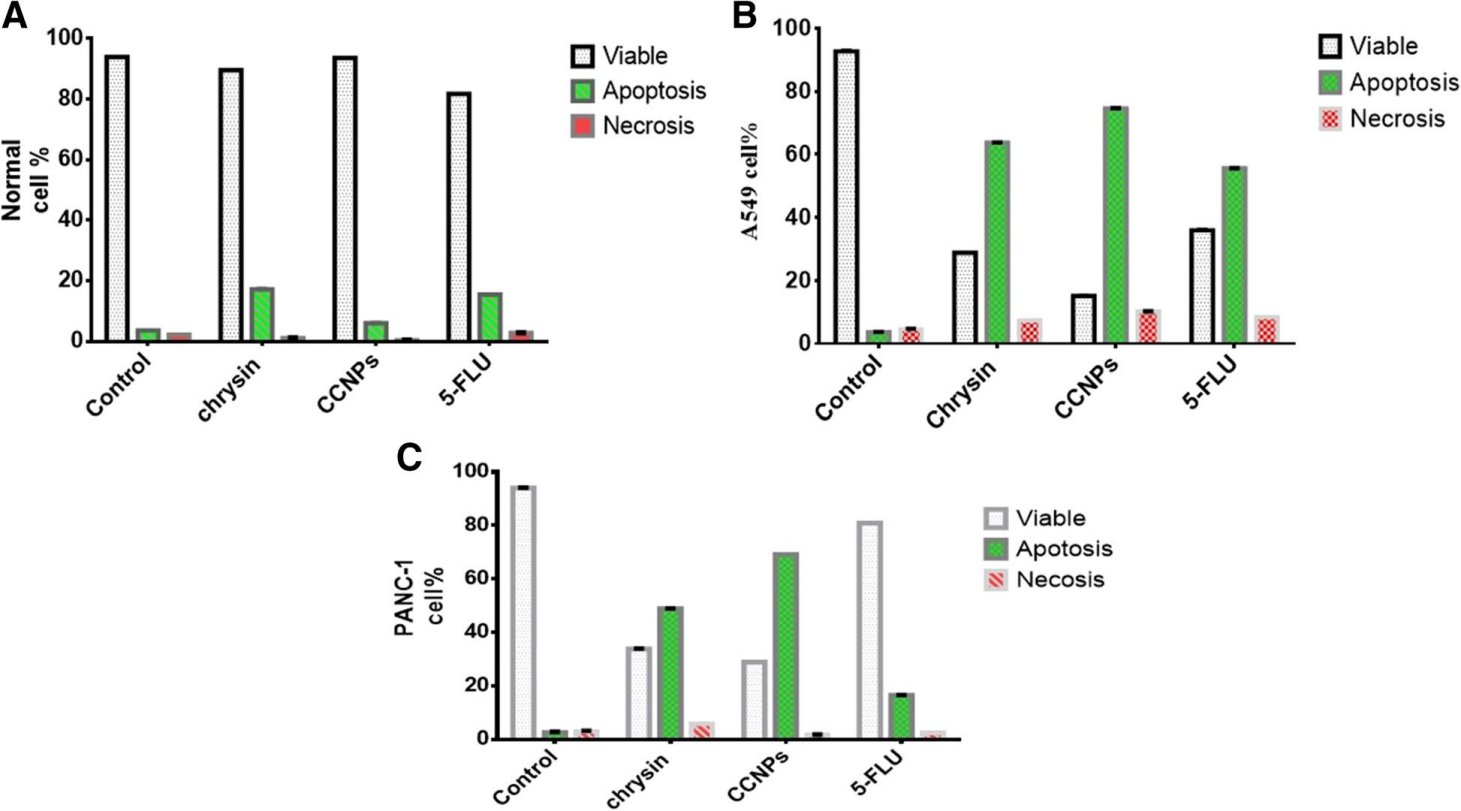
Graphical presentation of % of cells in each gate in (**a**) normal, (**b**) A549, and (**c**) PANC-1 cell lines after 48 h of incubation with IC_50_ concentration

The apoptotic and necrotic cell count was observed after treatment with IC_50_ concentrations of chrysin, CCNPs, or 5-FLU for 48 h. The apoptotic effect of chrysin, CCNPs, or 5-FLU was significantly increased in comparison with their necrotic effect. There was a significant apoptotic effect in A549 and PANC-1 cells (*p ≤ 0.001 and *p* ≤ 0.01, respectively) (CCNPs > chrysin > 5-FLU). There was a necrotic effect in A549 cells (CCNPs > chrysin > 5-FLU) and in PANC-1 (chrysin > 5-FLU > CCNPs), although there were no significant effect changes in non-cancerous cells (*p > 0.05) (Fig. 6).

### Molecular analysis

#### Effect of 48 h administration of chrysin, CCNPs, and 5-FLU on the relative expression of SDH C and D subunits in A549, PANC-1, and non-cancerous cells

**Fig. 7:**
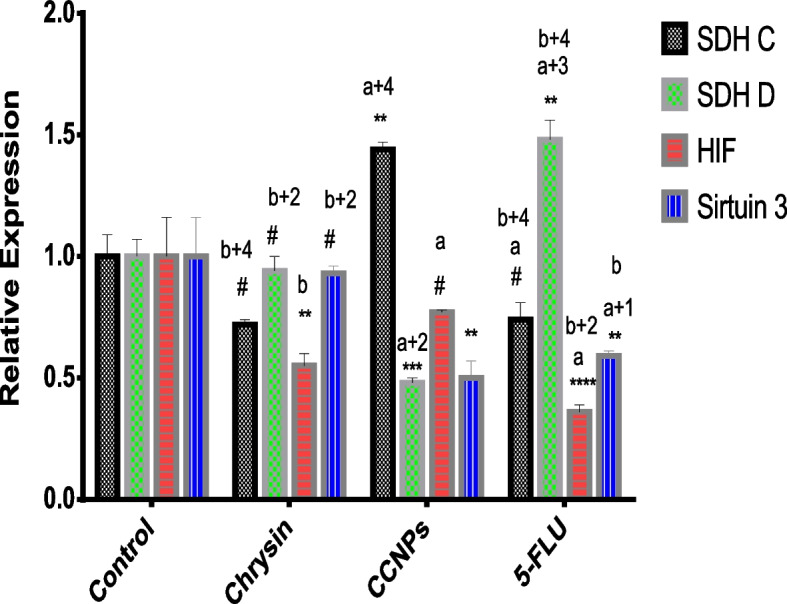
Effect of chrysin, CCNPs, and 5-FLU on the relative expression of SDH C, D, HIF, and SIRT-3 genes in normal cells after 48 h of incubation. Where data are shown as mean SEM (*n* = 3). A two-way ANOVA test was carried out. ** *P* < 0.01, ***P < 0.001 and ****P < 0.0001, # for non-significant versus the corresponding control group. While a^+1^ *p* < 0.05, a^+2^ *p* < 0.01, a^+3^ *p* < 0.001, a^+4^ *p* < 0.0001, a for non—significant versus the corresponding chrysin, b^+1^ *p* < 0.05, b^+2^ *p* < 0.01, b^+3^ *p* < 0.001, b^+4^ *p* < 0.0001, b for non- significant versus the corresponding CCNPs

**Fig. 8:**
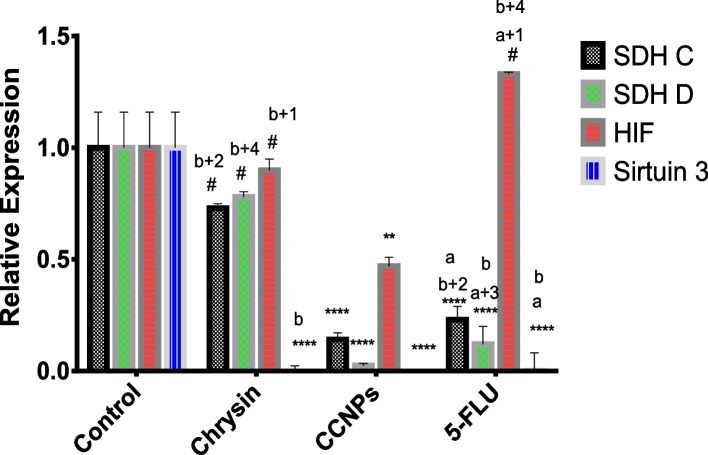
Effect of chrysin, CCNPs, and 5-FLU on the relative expression of SDH C, D, HIF, and SIRT-3 genes in A549 cells after 48 h of incubation. Where Data are shown as mean SEM (*n* = 3). A two-way ANOVA test was carried out. ** *P* < 0.01, ***P < 0.001 and ****P < 0.0001, # for non-significant versus the corresponding control group. While a^+1^ *p* < 0.05, a^+2^ *p* < 0.01, a^+3^ *p* < 0.001, a^+4^ *p* < 0.0001, a for non- significant versus the corresponding chrysin, b^+1^ *p* < 0.05, b^+2^ *p* < 0.01, b^+3^ *p* < 0.001, b^+4^ *p* < 0.0001, b for non- significant versus the corresponding CCNPs

**Fig. 9:**
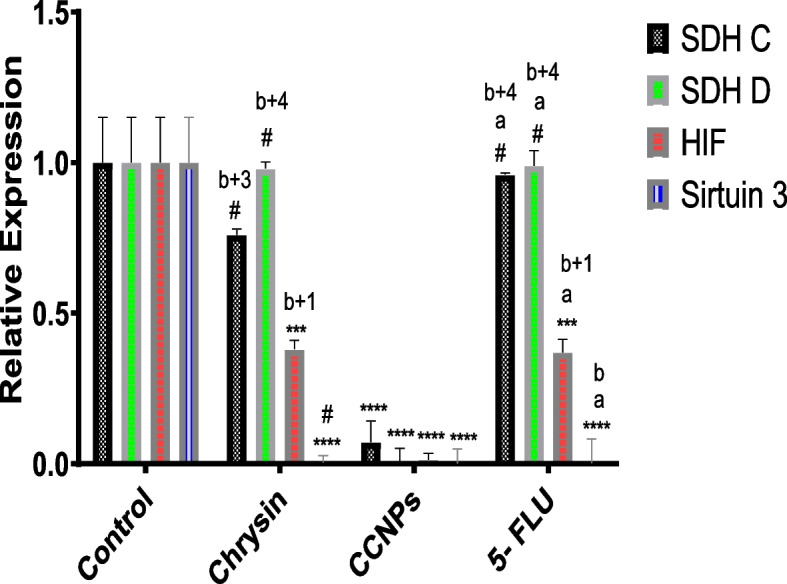
Effect of chrysin, CCNPs, and 5-FLU on the relative expression of SDH C, D, HIF, and sirtuin-3 genes in PANC-1cells after 48 h of incubation. Where data are shown as mean SEM (*n* = 3). A two-way ANOVA test was carried out. ** *P* < 0.01, ***P < 0.001 and ****P < 0.0001, # for non-significant versus the corresponding control group. While a^+1^ *p* < 0.05, a^+2^ *p* < 0.01, a^+3^ *p* < 0.001, a^+4^ *p* < 0.0001, a for non- significant versus the corresponding chrysin, b^+1^ *p* < 0.05, b^+2^ *p* < 0.01, b^+3^ *p* < 0.001, b^+4^ *p* < 0.0001, b for non- significant versus the corresponding CCNPs

There was a significant (*p ≤ 0.0001) downregulation of SDH C and D mRNA expression in A549 and PANC-1 cells following administration of chrysin, CCNPs, or 5-FLU at their IC_**50**_ concentration for 48 h compared with that in untreated cells. The lowest expression of SDH C and D mRNA was found in cells treated with CCNPs followed by that in cells treated with chrysin or 5-FLU (CCNPs < chrysin ≤ 5-FLU**)** with significant effect in non-cancerous cells (Figs. 7, 8 and 9).

#### Effect of 48 h administration of chrysin, CCNPs, and 5-FLU on the relative expression of HIF-1α in A549, PANC-1, and non-cancerous cell lines

HIF-1α is an essential transcript factor for the regulation of hypoxia-related genes. Our results revealed a significant (*p ≤ 0.0001) downregulation of HIF-1α mRNA expression in PANC-1 and A549 cells following administration of chrysin, CCNPs, or 5-FLU at their IC_50_ concentrations for 48 h as compared with that in untreated cells. The lowest expression was found in cells treated with CCNPs, followed by chrysin and 5-FLU (CCNPs < chrysin < 5-FLU) (Figs. 7, 8 and 9) although this was not shown in non-cancerous cells (CCNPs = 5-FLU < chrysin).

#### Effect of 48 h administration of chrysin, CCNPs, and 5-FLU on the relative expression of the SIRT-3 mRNA in A549, PANC-1, and non-cancerous cell lines

Sirtuins are class III histone deacetylases proteins, which have emerged as major non-histone protein acetylation regulators. Our results show a significant (*p ≤ 0.0001) downregulation of SIRT-3 mRNA expression l in A549 and PANC-1 cells following administration of chrysin, CCNPs, or 5-FLU at IC_50_ concentration for 48 h compared with that in untreated cells. The lowest expression was in the cells treated with CCNPs and 5-FLU followed by chrysin in PANC-1 and non-cancerous cells (CCNPs = 5-FLU < chrysin) and in A549 cells (CCNPs < chrysin < 5-FLU) (Fig. 7, 8 and 9).

## Discussion

PDAC and non-small lung tumors are predicted to be among the most common cancers globally between 2020 to 2030. A promising strategy for cancer therapy among those currently emerging is the targeting of tumor bioenergetics. The biochemical pathways that are involved in energy metabolism contribute to tumor initiation, survival, and resistance to therapy [38]. Mitochondrial complex ΙΙ (CΙΙ) functions not only in mitochondrial energy generation but also has a role in oxygen sensing. SDH differs from other mitochondrial dehydrogenases because of its unique redox properties. In partnership with ubiquinone, CII catalyzes the oxidation of succinate to fumarate as a step in the Krebs cycle. Another point of entrance for electrons is CII, which transfers them from succinate to CoQ via FeS clusters [66]. Therefore, we focused on inhibiting mitochondrial CΙΙ and ROS generation to induce apoptosis with arresting ATP generation in cancer cells. Combining these two approaches with natural treatments such as chrysin and their nanostructures is thus a promising cancer treatment option [20, 41]. The use of nanoparticles in medicine has received considerable attention recently because of improvements these bring to bioavailability, penetrative capacity, and treatment efficacy.

We recently performed a docking analysis that showed that the phenolic moiety of chrysin were tightly bonded to the active site of SDH by several hydrogen bonds, van der Waals, hydrophobic, and electrostatic bonds. The affinity of chrysin with SDH-like protein appeared to be caused by a putative conventional hydrogen bond with (VAL272, VAL296, ILE 183, ASP 203, ARG 298, THR 250, and LEU 274) and (GLY 181, PRO 182, CYS 249) at the active site side-chain. These results strongly suggest that SDH may interact with chrysin by forming a hydrogen link between the carbon-hydrogen chain (GLY 181, PRO 182, CYS 249) and inactive side-chain sites (VAL 272, VAL 296, ILE 183, ARG 203, ASP 203, ARG 298, THR 250, LEU 274), which would correlate with ubiquinone binding of SDH. The free binding energy was − 4.9, − 5, − 8.2, and − 8.4 kcal/mol [3, 33, 59, 61, 44, 57].

The technique of ionic gelation has enabled the manufacture of low-weight compounds with good encapsulation efficiency [58]. Our previous studies [44] showed a distinctive UV peak for chrysin at 348 nm. FT-IR analysis showed that the peaks of CCNPs include both chrysin and CS absorption bands. XRD analysis of CCNPs identified a peak shift that was attributed to the interaction between chrysin, CS, and trisodium polyphosphate (Tpp) which is in agreement with results in other studies [35, 53].

The zeta potential was found to be increased from + 35 to + 77.02 mV as shown in a previous study [16]. A positive zeta potential (+ 26 to + 39 mV) shows that particles have a high positive charge and these are likely to be more stable and possess mucoadhesion properties that enable them to permeate into tissues [40]. The nanoparticles were revealed to be spherical with an average particle size of 49.7 ± 3.02 nm. This size of the nanoparticle is better than the particle size of the previous study [58]. SEM investigation confirmed the spherical shape of the CCNPs, and this form is easier for cells to digest than a rod-shaped form [4]. Kinetic drug release of CCNPs was observed in the first 2 h followed by a constant release for up to 8 h, which agreed with the previous study that showed the drug kinetics of the loaded NPs initially underwent a burst release followed by a steady and sustainable release for 8 h, which then plateaus between 10 and 24 h [53, 44]. To cross the cell plasma membrane, cellular absorption of nanoparticles requires highly controlled systems with complicated biomolecular interactions. This biological membrane serves as a physical barrier that isolates the interior of a cell from its external environment. The plasma membrane has a general negative charge due to its structural and biomolecular properties phospholipid-based bilayer membrane dotted with proteins and other biomolecules [10, 9, 5]. Chitosan nanoparticles have a positive zeta potential, which improves drug delivery by facilitating attachment to negatively charged cell membranes and increases CCNPs stability. Also, TEM was used to confirm the particle size and shape. The spherical form can be more easily digested by cells.

In our recent study [44], the IC_50_ of chrysin, CCNPs, CNPs, and 5-FLU was evaluated in mitochondria isolated from normal adult mice at 34.66, 12.2, 184.1, and 0.05 μg/mL for SDH respectively. These IC_50_ values agreed with another study [51] that showed that the IC_50_ of chrysin ranged from 5–100 μM in liver rat mitochondria. Our results from the in vitro cytotoxicity study here gave IC_50_ values for chrysin of 57, 93, and 129.11 μg/mL in A549, PANC-1, and normal fibroblast cells, respectively. This agreed with previous studies [23, 44] that showed IC_50_ values of chrysin in different cancer cell lines of 38.7 to 49.2 μg/mL in A549 cells and 88.7 μg/mL for PANC-1 cells.

The IC_50_ values for CCNPs and CNPs were 14, 20, and 155.6 μg/mL and 394, 285, and 311 μg/mL in A549, PANC-1, and normal fibroblast cells, respectively, which agreed with results from previous studies [36, 1]. These results indicated that CCNPs could improve the efficiency of chrysin on cell growth inhibition [36]. The IC_50_ of 5-FLU was 2.8, 5.8, and 8.07 μg/mL in A549, PANC-1, and normal fibroblast cell lines, respectively [52, 54].

After the IC_50_ was determined, the activities of the C and D subunits of SDH were evaluated in different cell lines. Exposure to drugs at concentrations equal to their IC_50_ values caused a significant (*p* < 0.0001) increase in SDH inhibition as measured by the MTT assay. The sequence of inhibition was 5-FLU > chrysin > CCNPs for non-cancerous cells and chrysin > CCNPs > 5-FLU for A549 and PANC-1 cells**.** However, for SDH activity measured by DCPI assay, the sequence of inhibition was 5- FLU > chrysin > CCNP for non-cancerous cells and CCNPs > chrysin > 5- FLU for A549 and PANC-1 cells; treatment with CCNPs showed a significant (*p* < 0.0001) decrease compared with that with chrysin or 5-FLU.

SDH is known as CII in the mitochondrial respiratory chain complex, and CII acts as a direct link between the respiratory chain and the TCA cycle [6]. CII is the biomarker of mitochondrial inner membrane integrity, and its activity reflects the degree of mitochondrial activity. A reduction in CII activity has been reported to decrease the rates of mitochondrial respiration and ATP production [37].

Our results suggested that chrysin reduces SDH activity because of the high binding affinity of chrysin to CΙΙ subunits as theoretically shown via molecular docking. Chrysin can increase the generation of ROS through the disruption of CII activity, and our result agrees with those from a previous study [47, 51] that showed that chrysin strongly decreased SDH activity in CLL mitochondria. This effect could be explained by the structural and functional differences between mitochondria in normal and cancerous cells [47, 51].

Our results also showed that the CCNPs were more targeted to the ubiquinone site than chrysin, thereby improving the therapeutic and targeting effect. CCNPs also improved drug sensitivity in cancer cells with fewer side effects in non-cancerous cells compared with the effect of 5-FLU treatment [24].

We also assessed the effect of chrysin, CCNPs, and 5-FLU on the superoxide dismutase activity in cells. SOD, a vital cellular antioxidant, is heavily involved in the removal of O_2_. Superoxide anion free radical (O_2_-) is dismutated by these proteins into molecules of oxygen and hydrogen peroxide (H_2_O_2_) [64]. Excessive ROS provoke untoward events such as DNA damage and lipid membrane and protein peroxidation [19]. In addition, ROS are also thought to act as a potent mediator of apoptosis [28]. Our results showed that chrysin and CCNPs significantly (*p < 0.001) decreased SOD activity in a time-dependent manner in A549 and PANC-1 cells at their IC_50_ doses. This result agrees with a previous study that reported that inhibition and shifting UbQ frHom the membrane subunits of CII lead to the accumulation of electrons, that react with O_2_ and increase ROS to induce apoptosis [11, 6]. Our studies proved that chrysin and its targeted nanoparticles have a strong binding affinity to SDH especially the C and D subunits (ubiquinone site),inhibition of other mitochondria complexes leads to oxidation of succinate by SDH A and B subunits and the accumulation of electrons at the ubiquinone site react with O_2_ to produce superoxide free radicals in lung and pancreatic cancer cells [47, 51, 59–61, 66, 6, 14, 44, 49].

These results correlated with the significant increase in mitochondrial swelling in A549 and PANC-1 cells. CCNP treatment increased mitochondria swelling in the two cancer cell lines more than that induced by chrysin or 5-FLU (CCNPs > chrysin > 5-FLU). However, all the drugs induced significant changes in non-cancerous cells (*p ≤ 0.0001). We have shown that inhibition of SDH led to ROS accumulation, which then disrupted pore permeability in the inner mitochondrial membrane; this consequently increased solute uptake by mitochondria, leading to swelling of the mitochondrial matrix and also a break-up of the outer mitochondrial membrane. This enables soluble proteins (such as cytochrome c) to be released from the space between both membranes [51].

Apoptosis is a form of programmed cell death, which is a regular and controlled process in the growth and development of an organism; apoptosis is also known as cellular suicide because the cell participates in its demise. Here, we showed that the apoptotic effect of chrysin, CCNPs, and 5-FLU significantly increased in comparison with the necrotic effect. The sequence of apoptotic effect was CCNPs > chrysin > 5-FLU in A549 and PANC-1 cell lines, whereas the necrotic effect was CCNPs > chrysin > 5-FLU in A549 cells and chrysin > 5-FLU > CCNPs in PANC-1 cells. We suggest that CCNPs improved the apoptotic effect of chrysin with ROS formation causing oxidative stress and increasing permeability of mitochondria transition pores leading to pore opening and induction of apoptosis [47].

Molecular analysis demonstrated significant downregulation of SDH C and D mRNA expression in A549 and PANC-1 cells following administration of chrysin, CCNPs, or 5-FLU at the IC_**50**_ concentration for 48 h compared with that in untreated cells. The lowest expression of SDH C and D was in the two cancer cell lines treated with CCNPs followed by treatments with chrysin or 5-FLU on SDH C and D (CCNPs < chrysin ≤ 5-FLU**)**. The decreases in SDH C and D mRNA expression complement the inhibition of SDH C and D activity by CCNPs.

HIF-1α is an essential transcription factor for the regulation of hypoxia-related genes [48]. Several of these genes modulate molecular clusters attributed to the Warburg effect and associated pathways. Our results revealed a significant downregulation of HIF-1α mRNA expression in PANC-1 and A549 cells following the administration of chrysin, CCNPs, or 5-FLU at their IC_50_ concentration for 48 h compared with that in untreated cells. The lowest expression of HIF-1α mRNA was found in cells treated with CCNPs, followed by treatment with chrysin or 5-FLU (CCNPs < chrysin < 5-FLU), although this did not occur in non-cancerous cells (CCNPs = 5-FLU < chrysin). These results indicated that chrysin and CCNPs are potent inhibitors of HIF-1α expression and provide a new sight into their anticancer mechanisms. The inhibition of HIF-1α via multiple pathways can reduce tumorigenicity and angiogenesis that result from the inhibition of mitochondria CΙΙ [18, 6]*.*

Sirtuins are class III histone deacetylases proteins, which have emerged as major non-histone protein acetylation regulators. The mitochondrial SIRT-3 can affect mitochondrial energy production, oxidation of substrates, and apoptosis. SIRT-3 can regulate CΙΙ subunits (SDHA) activity [17, 6]. Our results revealed a significant downregulation of SIRT-3 mRNA expression in A549 and PANC-1 cells following the administration of chrysin, CCNPs, or 5-FLU at their IC_50_ concentration of IC_50_ for 48 h compared with that in untreated cells. The lowest SIRT-3 mRNA expression was in cells treated with CCNPs or 5-FLU followed by chrysin (CCNPs = 5-FLU < chrysin) in PANC-1 and non-cancerous cells, although in A549 cells both CCNP and chrysin were more effective than 5-FLU in decreasing expression (CCNPs < chrysin < 5-FLU). Recent studies have shown that by targeting a range of crucial regulators and their relevant pathways, SIRT-3 may perform the role of either a tumor suppressor or oncogene by affecting cell growth, leading to a decrease in cell proliferation. Accordingly, SIRT-3 has been shown to influence the biological behaviors of various tumors through different metabolic regulation patterns [63, 31]. From these results, these authors showed that chrysin and CCNPs were potent inhibitors not only of SDH but also the regulator SIRT-3 and could prevent tumor progress in A549 and PANC-1 cells. Finally, we recommend the trend towards natural products containing flavonoid compounds, such as chrysin and associated nanoparticles, that can treat various diseases, including cancer, without toxic side effects that result from using synthetic drugs.

## Conclusions

A diet that is rich in flavonoid-containing plants could indeed lead to reduced incidences of the more common types of cancer, such as pancreatic and lung cancers, that will be a major burden on global health by 2030–2035. Our study showed that CCNPs and chrysin were also likely to be powerful inhibitors of angiogenesis and tumorigenesis. Furthermore, we showed that CCNPs had more targeted action than chrysin at ubiquinone sites at CII subunits C and D, as indicated by molecular docking studies. Additionally, inhibition of succinate-ubiquinone oxidoreductase induced apoptosis because of the accumulation of electrons and increased superoxide formation with a significant decrease in superoxide dismutase activity as well as increased mitochondrial swelling in cancer cells. Molecular analysis showed that CCNPs significantly downregulated the expression of mRNA of the CΙΙ C and D subunits. Furthermore, CCNPs downregulated the expression of mRNA of SIRT-3, the most powerful regulator of SDH. CCNPs and chrysin also significantly downregulated the expression of an mRNA of HIF-1, whose activity in cells correlates with tumorigenicity and angiogenesis. We concluded that CCNPs improved the inhibitory effect of chrysin on succinate-coenzyme Q oxidoreductase expression and activity. In addition, both were more potent inhibitors of HIF-1 and SIRT-3 than 5-FLU, especially in pancreatic and lung cancers.

## Supplementary Information

**Additional file 1:** **Table 1.** The* in vitro* drug release profile of chrysin from the CCNPs. **Table 2.** Determination of succinate dehydrogenase (SDH) and Coenzyme Q reductase (complex II) activities by IC_50_. **Table 3.** Determination of succinate dehydrogenase (SDH) and Coenzyme Q reductase (complex II) activities by IC_50_ in normal fibroblast cell lines. **Table 4.** forward and reverse primers of 5 genes (1)- housekeeping gene GAPDH , (2,3) – SDH subunit C ,D. (4) – sirtuin-3 (5) –HIF. **Table 5.** Cytotoxic activity of chrysin on non-cancerous, A549, PANC-1 cell lines after 48h incubation. Results were expressed as (mean ± SEM, *n*=3). Table (6): IC_50_ of chrysin ,CCNPs, CNPs and 5-FLU on non-cancerous , A549 and PANC-1 cell lines for 48 h. **Table 7.** Effect of chrysin , CCNPs and 5-FLU on SDH activity by MTT Test in normal , A549 and PANC-1cancer cell lines. **Table 8.** Effect of chrysin and CCNPs on Succinate-coenzyme Q oxidoreductase activity by DCPI in non-cancerous cell lines , A549 and PANC-1 cancer cell lines. **Table 9.** Effect of 48h administration of chrysin CCNPs and 5-FLU on superoxide dismutase (SOD) activity in non-cancerous, A549 and PANC-1 cancer cell lines. **Table 10.** Effect of IC_50_ of chrysin, CCNPs, and 5-FLU on mitochondria swelling for 18 min in non-cancerous, A549 and PANC-1 cancer cell lines. **Table 11.** Percent of viable, apoptotic, necrotic, and dead (a) Normal, (b) A549, and (c) PANC-1 after treatment with IC_50_ chrysin, CCNPs and 5-FLU after 48 h incubation. **Table 12.** Effect of chrysin, CCNPs, and 5-FLU on the relative expression of SDH C, D, HIF, and sirtuin-3 genes in non-cancerous cells after 48h of incubation. **Table 13.** Effect of chrysin, CCNPs, and 5-FLU on the relative expression of SDH C, D, HIF, and sirtuin-3 genes in A549 cells after 48h of incubation. **Table 14.** Effect of chrysin, CCNPs, and 5-FLU on the relative expression of SDH C, D, HIF, and sirtuin-3 genes in PANC-1cell lines after 48h of incubation.

## Data Availability

All data generated or analysed during this study are included in this published article.

## Abbreviations

CLL: Chronic lymphocytic leukemia
PDAC: Pancreatic ductal adenocarcinoma
CS: Chitosan
CCNPs: Chrysin nanoparticles
5-FLU: 5-Fluorouracil
CII: Mitochondrial complex II
SDH: Succinate dehydrogenase
ROS: Reactive oxygen species
FT-IR: Fourier transform-infrared
XRD: X-ray powder diffraction
SEM: Scanning electron microscope
TEM: Transmission electron microscope
RT-qPCR: Reverse transcriptase quantitative PCR
SOD: Superoxide dismutase
SIRT-3: Sirtuin-3
HIF-1: Hypoxia-inducible factor 1
