# Supplementary material for: Impairment of electron transport chain and induction of apoptosis by chrysin nanoparticles targeting succinate-ubiquinone oxidoreductase in pancreatic and lung cancer cells
Source: Genes Nutr. 2023 Mar 11;18:4. doi: 10.1186/s12263-023-00723-4 (PMC10008604; doi:10.1186/s12263-023-00723-4)
Supplement: Supplementary file 1 — Additional file 1: Table 1. The in vitro drug release profile of chrysin from the CCNPs. Table 2. Determination of succinate dehydrogenase (SDH) and Coenzyme Q reductase (complex II) activities by IC50. Table 3. Determination of succinate dehydrogenase (SDH) and Coenzyme Q reductase (complex II) activities by IC50 in normal fibroblast cell lines. Table 4. forward and reverse primers of 5 genes (1)- housekeeping gene GAPDH , (2,3) – SDH subunit C ,D. (4) – sirtuin-3 (5) –HIF. Table 5. Cytotoxic activity of chrysin on non-cancerous, A549, PANC-1 cell lines after 48h incubation. Results were expressed as (mean ± SEM, n=3). Table (6): IC50 of chrysin ,CCNPs, CNPs and 5-FLU on non-cancerous , A549 and PANC-1 cell lines for 48 h. Table 7. Effect of chrysin , CCNPs and 5-FLU on SDH activity by MTT Test in normal , A549 and PANC-1cancer cell lines. Table 8. Effect of chrysin and CCNPs on Succinate-coenzyme Q oxidoreductase activity by DCPI in non-cancerous cell lines , A549 and PANC-1 cancer cell lines. Table 9. Effect of 48h administration of chrysin CCNPs and 5-FLU on superoxide dismutase (SOD) activity in non-cancerous, A549 and PANC-1 cancer cell lines. Table 10. Effect of IC50 of chrysin, CCNPs, and 5-FLU on mitochondria swelling for 18 min in non-cancerous, A549 and PANC-1 cancer cell lines. Table 11. Percent of viable, apoptotic, necrotic, and dead (a) Normal, (b) A549, and (c) PANC-1 after treatment with IC50 chrysin, CCNPs and 5-FLU after 48 h incubation. Table 12. Effect of chrysin, CCNPs, and 5-FLU on the relative expression of SDH C, D, HIF, and sirtuin-3 genes in non-cancerous cells after 48h of incubation. Table 13. Effect of chrysin, CCNPs, and 5-FLU on the relative expression of SDH C, D, HIF, and sirtuin-3 genes in A549 cells after 48h of incubation. Table 14. Effect of chrysin, CCNPs, and 5-FLU on the relative expression of SDH C, D, HIF, and sirtuin-3 genes in PANC-1cell lines after 48h of incubation. [file 12263_2023_723_MOESM1_ESM.docx]

**Table (1): The *in vitro* drug release proﬁle of chrysin from the CCNPs.**

| NO | Time (hr) | Drug Release % |
| --- | --- | --- |
| 1 | 2 |  |
| 2 | 4 | 28.86 ± 0.057 |
| 3 | 6 | 57.88 ± 0.0057 |
| 4 | 8 | 63.19 ± 0.0577 |
| 5 | 10 | 68.43 ± 0.0152 |
| 6 | 12 | 69.70 ± 0.035 |
| 7 | 14 | 78.9 ± 0.05 |
| 8 | 16 | 80.28 ± 0.02 |
| 9 | 18 | 81.55 ± 0.96 |
| 10 | 20 | 85.2 ± 1.039 |
| 11 | 22 | 85.2± 1.03 |
| 12 | 24 | 85.2 ± 1 |

**Table (2): Determination of succinate dehydrogenase (SDH) and Coenzyme Q reductase (complex II) activities by IC_50_.**

| Relative Enzyme Activity % | Negative control  (untreated cells) | Chrysin  mean± SEM  **p* value | CNPs  mean± SEM  **p* value | CCNPs  mean± SEM  **p* value | 5-fluorouracil mean± SEM  **p* value | |
| --- | --- | --- | --- | --- | --- | --- |
| Succinate Dehydrogenase by MTT test | 100 ± 0 | 40.14±0.03  *P* <0.0001 | 90.9± 0.005  *P* <0.0001 | 86.7±0.041  *P* <0.0001 | 89± 0.02  *P* <0.0001 |  |
| Ubiquinone Reductase by DCPI dye | 100 ± 0 | 70.90±0.003  *P* <0.0001 | 86.74 ± 0.005    *P* <0.0001 | 60.8 ±0.002  *P* <0.0001 | 80.23 ±0.02  *P* < 0.0001 |  |

****p* value versus the negative control (untreated cells)**

**Table (3): Determination of succinate dehydrogenase (SDH) and Coenzyme Q reductase (complex II) activities by IC_50_ in normal fibroblast cell lines.**

| Relative Enzyme Activity % | Negative control  (untreated cells) | Chrysin  mean± SEM  **p-*value | CCNPs  mean± SEM  **p* value | 5-fluorouracil mean± SEM  **p* value | |
| --- | --- | --- | --- | --- | --- |
| Succinate Dehydrogenase by MTT test | 100 ± 0 | 80.90 ±0.003  *P* <0.0001 | 89.06 ±0.0003  *P* <0.0001 | 90± 0.003  *P* <0.0001 |  |
| Ubiquinone Reductase by DCPI dye | 100 ± 0 | 90±0.002  *P* <0.0001 | 85±0.02  *P* <0.0001 | 95 ±0.001  *P* < 0.0001 |  |

****p* value versus the negative control (untreated cells)**

**Table (4): forward and reverse primers of 5 genes (1)- housekeeping gene GAPDH , (2,3) – SDH subunit C ,D. (4) – sirtuin-3 (5) –HIF**

| Gene | Forward primer  (^/^5 ------ ^/^3) | Reverse primer  (^/^5 ------ ^/^3) | Size  (bp) |
| --- | --- | --- | --- |
| *GAPDH* | GATTCCACCCATGGCAAATTC | CTGGAAGATGGTGATGGGATT | 87 |
| *SDHC* | GATGGAGCGGTTCTGGAATAA | CATGGGAAGAGACCAACTGTAG | 85 |
| *SDHD* | CATTTCTTCAGGACCGACCTATC | AACTTGTCCAAGGCCCAAT | 88 |
| *Sirtuin3* | AGGGACGATGATGTAGCTGA | GGCGATCTGAAGTCTGGAATG | 110 |
| *HIF* | GTCTGCAACATGGAAGGTATTG | GCAGGTCATAGGTGGTTTCT | 103 |

**Table (5):** Cytotoxic activity of chrysin on non-cancerous, A549, PANC-1 cell lines after 48h incubation. Results were expressed as (mean ± SEM, n=3)

**(A)**

| **Concentration of**  **Chrysin (μM)** | **%Viability of**  **normal fibroblast** | **%Viability**  **of A549** | **%Viability**  **of PANC-1** |
| --- | --- | --- | --- |
| **Negative control**  **(untreated cells)** | 100 ± 0 | 100 ± 0 | 100 ± 0 |
| **12.5**  ****p* value** | 99.76423±0.012  *p*<0.01 | 98.14±0.014  *p*<0.01 | 99.04±*0.025 p*<0.01 |
| **25**  ****p* value** | 90.116±0.013  *p*<0.01 | 93.06 ±0.01  *p*<0.01 | 99.00±0.013  *p*<0.01 |
| **50**  ****p* value** | 89.42 ±0.004  *p*<0.01 | 87.49±0.02  *p*<0.01 | 89.5 ±0.00849  *p*<0.01 |
| **100**  ****p* value** | 84.17 ±0.006  *p*<0.01 | 72.95±0.007  *p*<0.01 | 89.13 ±0.011  *p*<0.01 |
| **200**  ****p* value** | 72.6 ±0.006  *p*<0.01 | 43.99±0.002  *p*<0.01 | 32.02 ±0.0295  *p*<0.01 |

**(B)**

| **Concentration of**  **CCNPs (μM)** | **%Viability**  **of normal fibroblast** | **%Viability**  **of A549** | **%Viability**  **of PANC-1** |
| --- | --- | --- | --- |
| **Negative control**  **(untreated cells)** | 100 ± 0 | 100 ± 0 | 100 ± 0 |
| **12.5**  ****p* value** | 99 ±0.01  *p*<0.01 | 71.13±0.0074 *p*<0.01 | 99 ±*0.025*  *P<0.01* |
| **25**  ****p* value** | 93.10±0.016  *p*<0.01 | 59.55±0.021 *p*<0.01 | 89.13±0.06 *p*<0.01 |
| **50**  ****p* value** | 91.95±0.011  *p*<0.01 | 57.91±0.010 *p*<0.01 | 89±0.021 *p*<0.01 |
| **100**  ****p* value** | 86.69 ±0.0.009  *p*<0.01 | 37.92±0.001 *p*<0.01 | 62±0.025 *p*<0.01 |
| **200**  ****p* value** | 75.46 ±*0.003*  *p*<0.01 | 30.55±0.0142 *p*<0.01 | 32.31±0.006 *p*<0.01 |

****p* value versus the negative control (untreated cells)**

**(C)**

| **Concentration of**  **CNPs (μg/mL)** | **%Viability**  **of normal** | **%Viability**  **of A549** | **%Viability**  **of PANC-1** |
| --- | --- | --- | --- |
| **Negative control**  **(untreated cells)** | 100 ± 0 | 100 ± 0 | 100 ± 0 |
| **500**  ****p* value** | 43.51 ±0.12  *p*<0.01 | 67.87±0.0074  *p*<0.01 | 53.22 ±*0.013 p*<0.01 |
| **1000**  ****p* value** | 15.75 ±0.003 *p*<0.01 | 6.43 ±0.021  *p*<0.01 | 4.64 ±0.007  *p*<0.01 |
| **2000**  ****p* value** | 15 ±0.0008  *p*<0.01 | 6.1 ±0.010  *p*<0.01 | 4.3 ±0.011  *p*<0.01 |

**(D)**

| **Concentration of**  **5-FLU (μM)** | **%Viability**  **of normal** | **%Viability**  **of A549** | **%Viability**  **of PANC-1** |
| --- | --- | --- | --- |
| **Negative control**  **(untreated cells)** | 100 ± 0 | 100 ± 0 | 100 ± 0 |
| **3.13**  ****p* value** | 94.11±0.01  *p*<0.01 | 95.71±0.12  *p*<0.01 | 78.7±0.13  *p*<0.01 |
| **12.5**  ****p* value** | 91.4±0.01  *p*<0.01 | 69.16±0.0074  *p*<0.01 | 63.6 ±*0.01*  *p*<0.03 |
| **50**  ****p* value** | 74.31±0.013  *p*<0.01 | 61.99±0.002  *p*<0.01 | 41.84±0.2  *p*<0.01 |
| **100**  ****p* value** | 13.92 ±0.043  *p*<0.01 | 6.73 ±0.0010  *p*<0.01 | 5.13±0.05  *p*<0.01 |

****p* value versus the negative control (untreated cells)**

**Table (6): IC_50_ of chrysin ,CCNPs, CNPs and 5-FLU on non-cancerous , A549 and PANC-1 cell lines for 48 h.**

| **Ic_50_ Concentration of treatment (μg/mL)** | **Cell lines** | | |
| --- | --- | --- | --- |
|  | **Normal** | **A549** | **PANC-1** |
| **Negative control**  **(untreated cells)** | 100 ± 0 | 100 ± 0 | 100 ± 0 |
| **Chrysin**  **mean± SEM**  ****p* value** | 129 ±0.00849  *p*<0.01 | 57±0.02  *p*<0.01 | 93±0.004  *p*<0.01 |
| **CCNPs**  **mean± SEM**  ****p* value** | 156±0.00849  *p*<0.01 | 14 ±0.014  *p*<0.01 | 20 ±0.012  *p*<0.01 |
| **5 FLU**  **mean± SEM**  ****p* value** | 8.07 ±*0.025 p*<0.01 | 5.8±0.05  *p*<0.01 | 2.8 ±0. 13  *p*<0.01 |
| **CNPs**  **mean± SEM**  ****p* value** | 311.1 ±0.013  *p*<0.01 | 394±0. 05  *p*<0.001 | 285 ±0. 005  *p*<0.0001 |

****p* value versus the negative control (untreated cells).**

**Table (7): Effect of chrysin , CCNPs and 5-FLU on SDH activity by MTT Test in normal , A549 and PANC-1cancer cell lines.**

| **Treatment/**  **Relative Activity %** | **MTT SDH activity (nmole/min/mg) in different cell lines** | | |
| --- | --- | --- | --- |
|  | **Normal fibroblast**  **mean± SEM**  ***P Value*** | **A549**  **mean± SEM**  ***P Value*** | **PANC-1**  **mean± SEM**  ***P Value*** |
| **Untreated**  **mean± SEM**  ***P* Value** | 1.15956± 0.00014  **P* <0.0001 | 0.77240 ± 0.000333  **P* <0.0001 | 0.446000±0.00033  **P* <0.0001 |
| **Relative Activity%** | 100 | 100 | 100 |
| **Inhibition %** | 0 | 0 | 0 |
| **Chrysin**  **mean± SEM**  ***P* Value** | 0.79 ± 0.00019  **P*<.0001 | 0.4 ± 0.1  **P*<0.0001 | 0.2 ± 0.5  **P*<0.0001 |
| **Relative Activity%** | 71.8 | 51.9 | 45.45 |
| **Inhibition %** | 28.2 | 48.1 | 54.55 |
| **CCNPs**  **mean± SEM**  ***P* Value** | 0.8 ±0.1  **P*<0.0001 | 0.45 ± 0.23  **P*<0.0001 | 0.28 ±0.2  **P*<0.0001 |
| **Relative Activity%** | 72.7 | 62.3 | 63.6 |
| **Inhibition%** | 27.3 | 37.7 | 36.4 |
| **5-fluorouracil**  **mean± SEM**  ***P Value*** | 0.7 ± 0.5  **P*<0.0001 | 0.5 ± 0.2  **P*<0.0001 | 0.3 ± 0.31  **P*<0.0001 |
| **Relative Activity%** | 63.6 | 64.9 | 68.1 |
| **Inhibition%** | 36.4 | 35.1 | 31.9 |

****p* value versus the negative control (untreated cell)**

**Table (8): Effect of chrysin and CCNPs on Succinate-coenzyme Q oxidoreductase activity by DCPI in non-cancerous cell lines , A549 and PANC-1**

**cancer cell lines.**

| **Treatment /Relative Activity %** | **Succinate-co enzyme Q oxidoreductase activity (nmol/min/mg) in different cell lines** | | |
| --- | --- | --- | --- |
|  | **Non-cancerous cell** | **A549** | **PANC-1** |
| ***Untreated***  **mean± EM**  ***P Value*** | 0.2 ± 0.0033   \| **P <0.0001* \| \| --- \| \|  \| \|  \| | 0.0443 ± 0.0017  **P <0.0001* | 0.066 ± 0.0039  **P <0.0001* |
| **Relative Activity%** | \| 100 \| \| --- \| \|  \| | 100 | 100 |
| **Inhibition%** | 0 | 0 | 0 |
| **Chrysin**  **mean± SEM**  ***P* Value** | 0.1± 0.1  **P*<0.0001 | 0.01 ± 0.5  **P*<0.0001 | 0.0077 ± 0.0097  **P*<0.0001 |
| **Relative Activity%** | 50 | 25 | 11.6 |
| **Inhibition%** | 50 | 75 | 88.4 |
| **CCNPs**  **mean± SEM**  ***P* Value** | 0.13± 0.23  **P<.0001* | 0.007±0.4  **P<.0001* | 0.002 ±0.3  **P<.0001* |
| **Relative Activity**  **%** | 65 | 17.5 | 3.33 |
| **Inhibition%** | 35 | 82.5 | 96.67 |
| **5-fluorouracil**  **mean± SEM**  ***P Value*** | 0.07± 0.1  **P*<0.0001 | 0.023 ±0.00033  **P*<0.0001 | 0.008±0.11  **P*<0.0001 |
| **Relative Activity**  **%** | 35 | 57.5 | 13.33 |
| **Inhibition%** | 65 | 42.5 | 86.67 |

****p* value versus the negative control (untreated cells).**

**Table. (9): Effect of 48h administration of chrysin CCNPs and 5-FLU on superoxide dismutase (SOD) activity in non-cancerous, A549 and PANC-1 cancer cell lines.**

| **Treatment** | **SOD activity (IU/ml) for 48 h** **in different cell lines** | | |
| --- | --- | --- | --- |
|  | **Normal fibroblast** | **A549** | **PANC-1** |
| **untreated**  **mean± SEM**  ***P Value*** | 347.30±0.82  *P*<0.0001 | 328.67±0.69  *P*<0.001 | 325.136±0.342  *P*<0.0001 |
| **Chrysin**  **mean± SEM**  ***P Value*** | 340.92±0.55  *P*<0.0001 | 170.05±0.36  *P*<0.001 | 155.66±0.50  *P*<0.0001 |
| **CCNPs**  **mean± SEM**  ***P Value*** | 349.90±0.55  *P*<0.0001 | 130.69±0.52  *P*<0.0001 | 110.45±0.57  *P*<0.0001 |
| **5-FLU**  **mean± SEM**  ***P Value*** | 320.19±0.67  *P*<0.0001 | 211.02±0.66  *P*<0.0001 | 200.16±2.07  *P*<0.0001 |

****p* value versus the negative control (untreated cells).**

**Table (10): Effect of IC_50_ of chrysin, CCNPs, and 5-FLU on mitochondria swelling for 18 min in non-cancerous, A549 and PANC-1 cancer cell lines.**

| IC_50_ of treatment | Normal | | A549 | | PANC-1 | |
| --- | --- | --- | --- | --- | --- | --- |
|  | **Time (min)** | **Absorbance**  **(540nm)** | **Time**  **(min)** | **Absorbance**  **(540nm)** | **Time (min)** | **Absorbance**  **(540nm)** |
| Control  mean±SEM  *P value* | **0** | 0.346±0.006  **P*=0.0001 | 0 | 1.65±0.02  **P*=0.0001 | 0 | 1.2±0.06  **P*=0.000 |
|  | **3** | 0.347±0.002  **P*=0.0001 | 3 | 1.55±0.02  **P*=0.0001 | 3 | 0.98±0.006  **P*=0.0001 |
|  | **6** | 0.341±0.0007  **P*=0.0001 | 6 | 1.67±0.007  **P*=0.0001 | 6 | 0.96±0.003  **P*=0.0001 |
|  | **9** | 0.32±0.001  **P*=0.0001 | 9 | 0.92±0.017  **P*=0.0001 | 9 | 0.92±0.017  **P*=0.0001 |
|  | **12** | 0.29±0.001  **P*=0.0001 | 12 | 0.8 6±0.03  **P*=0.0001 | 12 | 0.80±0.001  **P*=0.0001 |
|  | **15** | 0.271±0.001  **P*=0.0001 | 15 | 0.88±0.02  **P*=0.0001 | 15 | 0.84±0.03  **P*=0.0001 |
|  | **18** | 0.27±0.001  **P*=0.0001 | 18 | 0.16±0.01  **P*=0.0001 | 18 | 0.27±0.001  **P*=0.0001 |
| Chrysin  mean±SEM  *P value* | **0** | 0.31±0.003  **P*=0.0001 | 0 | 1.62±0.02  **P*=0.0001 | 0 | 1.26±0.06  **P*=0.0001 |
|  | **3** | 0.296±0.001  **P*=0.0001 | 3 | 1±0.06  **P*=0.0001 | 3 | 0.98±0.03  **P*=0.0001 |
|  | **6** | 0.248±0.001  **P*=0.0001 | 6 | 0.85±0.04 | 6 | 0.81±0.01  **P*=0.0001 |
|  | **9** | 0.23±0.003  **P*=0.0001 | 9 | 0.71±0.01  **P*=0.0001 | 9 | 0.65±0.008  **P*=0.0001 |
|  | **12** | 0.22±0.001  **P*=0.0001 | 12 | 0.61±0.003  **P*=0.0001 | 12 | 0.61±0.003  **P*=0.0001 |
|  | **15** | 0.189±0.003  **P*=0.0001 | 15 | 0.57±0.003  **P*=0.0001 | 15 | 0.57±0.003  **P*=0.0001 |
|  | **18** | 0.16±0.01  **P*=0.0001 | 18 | 0.16±0.01  **P*=0.0001 | 18 | 0.16±0.01  **P*=0.0001 |
| CCNPs  mean±SEM  *P value* | **0** | 0.31±0.003  **P*=0.0001 | 0 | 1.65±0.01  **P*=0.0001 | 0 | 1.28±0.07  **P*=0.0001 |
|  | **3** | 0.297±0.0008  **P*=0.0001 | 3 | 0.82±0.05  **P*=0.0001 | 3 | 0.82±0.05  **P*=0.0001 |
|  | **6** | 0.24±0.004  **P*=0.0001 | 6 | 0.67±0.06  **P*=0.0001 | 6 | 0.67±0.006  **P*=0.0001 |
|  | **9** | 0.23±0.003  **P*=0.0001 | 9 | 0.53±0.01  **P*=0.0001 | 9 | 0.54±0.01  **P*=0.0001 |
|  | **12** | 0.21±0.001  **P*=0.0001 | 12 | 0.44±0.01  **P*=0.0001 | 12 | 0.44±0.01  **P*=0.0001 |
|  | **15** | 0.18±0.003 | 15 | 0.35±0.01  **P*=0.0001 | 15 | 0.35±0.01  **P*=0.0001 |
|  | **18** | 0.19±0.001 | 18 | 0.19±0.001  **P*=0.0001 | 18 | 0.19±0.001  **P*=0.0001 |
| 5-FLU  mean±SEM  *P value* | **0** | 0.314±0.001  **P*=0.0001 | 0 | 1.17±0.29  **P*=0.0001 | 0 | 1.2±0.03  **P*=0.0001 |
|  | **3** | 0.29±0.0006  **P*=0.0001 | 3 | 0.98±0.008  **P*=0.0001 | 3 | 0.98±0.008  **P*=0.0001 |
|  | **6** | 0.25±0.001  **P*=0.0001 | 6 | 0.86±0.01  **P*=0.0001 | 6 | 0.86±0.01  **P*=0.0001 |
|  | **9** | 0.22±0.002  **P*=0.0001 | 9 | 0.82±0.01  **P*=0.0001 | 9 | 0.82±0.01  **P*=0.0001 |
|  | **12** | 0.238±0.008  **P*=0.0001 | 12 | 0.72±0.01  **P*=0.0001 | 12 | 0.72±0.012  **P*=0.0001 |
|  | **15** | 0.146±0.01  **P*=0.0001 | 15 | 0.66±0.01  **P*=0.0001 | 15 | 0.66±0.01  **P*=0.0001 |
|  | **18** | 0.146±0.01  **P*=0.0001 | 18 | 0.146±0.01  **P*=0.0001 | 18 | 0.14±0.01  **P*=0.0001 |

****p value* : versus non treated cells.**

**Table (11): percent of viable, apoptotic, necrotic, and dead (a) Normal, (b) A549, and (c) PANC-1 after treatment with IC_50_ chrysin, CCNPs and 5-FLU after 48 h incubation.**

**a**

| Ic_50_ Concentration of Treatment  (μg/mL) | Normal cell lines | | |
| --- | --- | --- | --- |
|  | **Viable** | **Apoptosis** | **Necrosis** |
| Negative control  (untreated cells) | **100 ± 0** | **100 ± 0** | **100 ± 0** |
| Chrysin  mean± SEM  **p* value | **89.61 ±0.012**  ****p*<0.001** | **17.26 ±0.014**  ****p*<0.0001** | **1.09 ±*0.025 *p*<0.0001** |
| CCNPs  mean± SEM  **p* value | **93.575 ±0. 13**  ***p*<0.001** | **6.02 ±0.05**  ****p*<0.001** | **0.405 ±0.013**  ****p*<0.001** |
| 5 FLU  mean± SEM  **p* value | **81.75 ±0.004**  ****p*<0.001** | **20.46 ±0.02**  ****p*<0.001** | **2.785 ±0.00849**  **** p*<0.001** |

**b**

| Ic_50_ Concentration of treatment  (μg/mL) | A549 cell lines | | |
| --- | --- | --- | --- |
|  | Viable | Apoptosis | Necrosis |
| Negative control  (untreated cells) | 100 ± 0 | 100 ± 0 | 100 ± 0 |
| Chrysin  mean± SEM  **p* value | 28.89 ±0.004  **p*<0.01 | 63.755 ±0.02  **p*<0.01 | 7.36 ±0.00849  **p*<0.01 |
| CCNPs  mean± SEM  **p* value | 15.07±0.012  **p*<0.01 | 74.6 ±0.014  **p*<0.01 | 10.28 ±*0.025*  **p*<0.01 |
| 5 FLU  mean± SEM  **p* value | 35.93 ±0. 13  **p*<0.01 | 55.61 ±0.05  **p*<0.01 | 8.46 ±0.013  **p*<0.01 |

C

| Ic_50_ Concentration of treatment  (μg/mL) | PANC-1 cell lines | | |
| --- | --- | --- | --- |
|  | Viable | Apoptosis | Necrosis |
| Negative control  (untreated cells) | 100 ± 0 | 100 ± 0 | 100 ± 0 |
| Chrysin  mean± SEM  **p* value | 33.85 ±0. 13  **p*<0.01 | 48.88 ±0.05  **p*<0.01 | 5.9 ±0.013  **p*<0.01 |
| CCNPs  mean± SEM  **p* value | 28.97 ±0.012  **p*<0.01 | 69.21 ±0.014  **p*<0.01 | 1.815 ±*0.025*  **p*<0.01 |
| 5 FLU  mean± SEM  **p* value | 80.93 ±0.004  **p*<0.05 | 16.57 ±0.02  **p*<0.05 | 2.485 ±0.008  **p*<0.05 |

****p value*: versus non treated cells.**

**Table (12): Effect of chrysin, CCNPs, and 5-FLU on the relative expression of SDH C, D, HIF, and sirtuin-3 genes in non-cancerous cells after 48h of incubation.**

| Ic_50_ Concentration of treatment  (μg/mL) |  |  |  |  |
| --- | --- | --- | --- | --- |
|  | **Fold of change in Normal cell line** | | | |
|  | **Subunit C** | **Subunit D** | **HIF** | **Sirtuin 3** |
| Control  Mean ± SEM  *P Value* | **1±0.09** | **1±0.07** | **1±0.16** | **1±0.16** |
| Chrysin  Mean ± SEM  *P Value* | **0.71 ± 0.02**  ****P<0.0001*** | **0.948±0.06**  ****P<0.001*** | **0.55 ±0.05**  ****P<0.0001*** | **0.93±0.03**  ****P<0.0001*** |
| CCNPs  Mean ± SEM  *P Value* | **1.44±0.03**  ****P<0.0001*** | **0.48 ±0.021**  ****P<0.001*** | **0.77±0.017**  ****P<0.0001*** | **0.505±0.07**  ****P<0.0001*** |
| 5 FLU  Mean ± SEM  *P Value* | **0.74 ±0.07**  ****P<0.0001*** | **1.48±0.081**  ****P<0.001*** | **0.36 ±0.03**  ****P<0.0001*** | **0.590±0.02**  ****P<0.0001*** |

****p value* : versus non-treated normal cells.**

**Table (13): Effect of chrysin, CCNPs, and 5-FLU on the relative expression of SDH C, D, HIF, and sirtuin-3 genes in A549 cells after 48h of incubation.**

| Ic_50_ Concentration of treatment  (μg/mL) |  |  |  |  |
| --- | --- | --- | --- | --- |
|  |  |  |  |  |
|  | **Fold of change in A549 cell lines** | | | |
|  | **Subunit C** | **Subunit D** | **HIF** | **Sirtuin 3** |
| Control  Mean ± SEM  *P Value* | **1±0.16** | **1±0.16** | **1±0.16** | **1±0.16** |
| Chrysin  Mean ± SEM  *P Value* | **0.729 ±0.02**  ****P*<0.0001** | **0.78±0.02**  ****P*<0.0001** | **0.9±0.05**  ****P*<0.0001** | **0.0001±0.023**  ****P*<0.0001** |
| CCNPs  Mean ± SEM  *P Value* | **0.141±0.03**  ****P*<0.0001** | **0.024±0.01**  ****P*<0.0001** | **0.47±0.04**  ****P*<0.0001** | **5.8X10^-3^  ±0.0001**  ****P*<0.0001** |
| 5 FLU  Mean ± SEM  *P Value* | **0.23±0.06**  ****P*<0.0001** | **0.12±0.08**  ****P*<0.0001** | **1.33±0.008**  ****P*<0.0001** | **0.002±0.08**  ****P*<0.0001** |

****p value* versus non-treated A549 cells.**

**Table (14): Effect of chrysin, CCNPs, and 5-FLU on the relative expression of SDH C, D, HIF, and sirtuin-3 genes in PANC-1cell lines after 48h of incubation.**

| Ic_50_ Concentration of treatment  (μg/mL) |  |  |  |  |
| --- | --- | --- | --- | --- |
|  | **Fold of change in PANC-1 cell lines** | | | |
|  | **Subunit C** | **Subunit D** | **HIF** | **Sirtuin 3** |
| Control  Mean ± SEM  *P Value* | **1±0.15** | **1±0.15** | **1±0.15** | **1±0.15** |
| Chrysin  Mean ± SEM  *P Value* | **0.96±0.030**  ****P*<0.0001** | **4.675±0.02**  ****P*<0.0001** | **0.386±0.03**  ****P*<0.0001** | **0.0036±0.023**  ****P*<0.0001** |
| CCNPs  Mean ± SEM  *P Value* | **0.072±0.073**  ****P*<0.0001** | **0.009±0.042**  ****P*<0.0001** | **0.0132±0.02**  ****P*<0.0001** | **0.0013±0.04**  ****P*<0.0001** |
| 5 FLU  Mean ± SEM  *P Value* | **1.185±0.006**  ****P*<0.0001** | **1.017±0.05**  ****P*<0.0001** | **0.37±0.04**  ****P*<0.0001** | **0.0013±0.089**  ****P*<0.0001** |

****p value* : versus non treated PANC-1 cells.**
